# Supplementary material for: Causal interplay between lactose intolerance and gut microbiota: a combined bidirectional Mendelian randomization and in vivo validation study
Source: Front Nutr. 2026 Jun 1;13:1803337. doi: 10.3389/fnut.2026.1803337 (PMC13265576; doi:10.3389/fnut.2026.1803337)
Supplement: Supplementary file 1 [file Data_Sheet_1.zip › supplementary materials/Forward/forest plot/ebi-a-GCST90027662.finngen_R12_E4_LACTONAS.pdf]

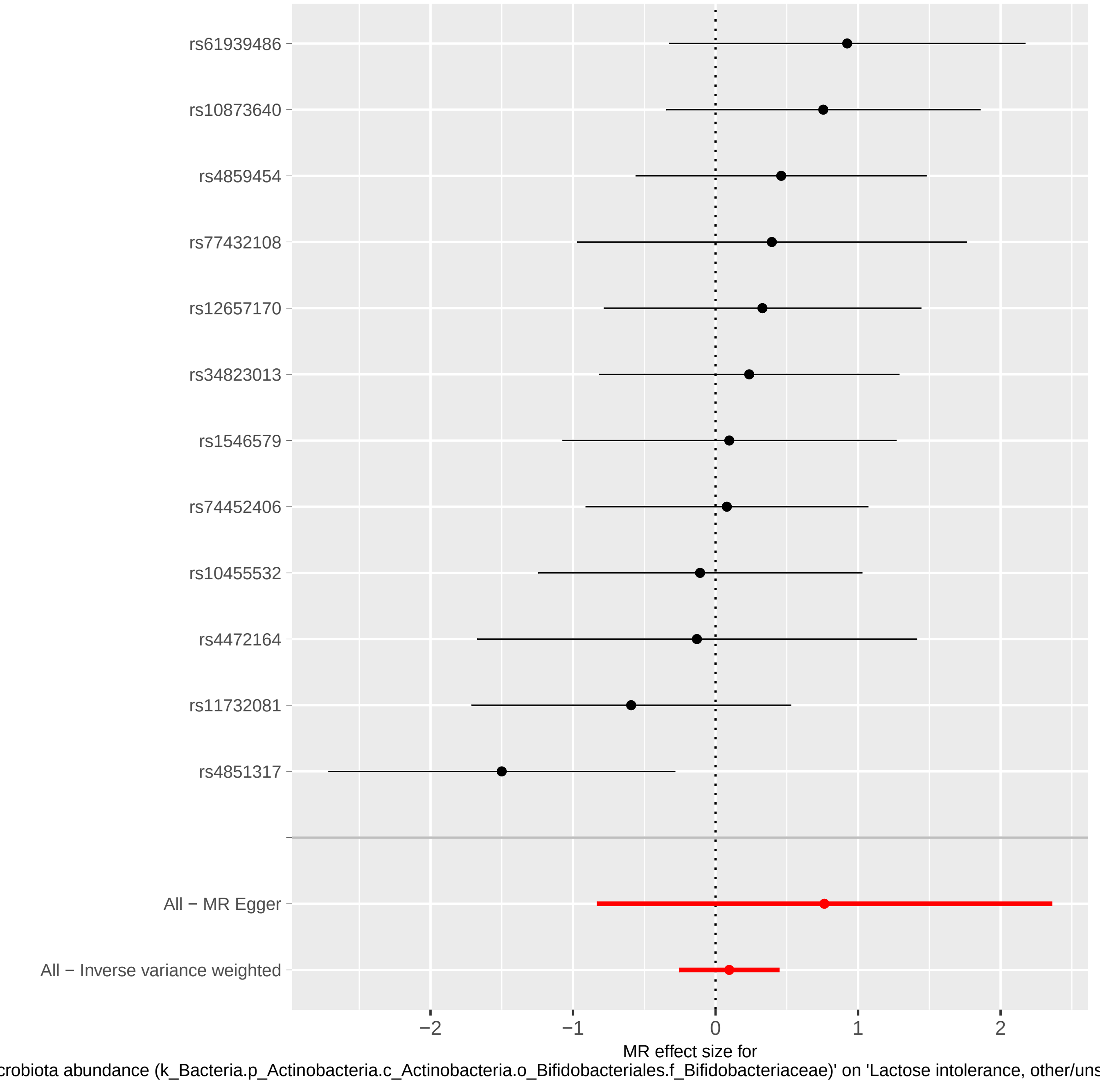

rs61939486
rs10873640
rs4859454
rs77432108
rs12657170
rs34823013
rs1546579
rs74452406
rs10455532
rs4472164
rs11732081
rs4851317
All − MR Egger
All − Inverse variance weighted
−2
−1
0
1
2
MR effect size for
crobiota abundance (k_Bacteria.p_Actinobacteria.c_Actinobacteria.o_Bifidobacteriales.f_Bifidobacteriaceae)' on 'Lactose intolerance, other/uns
